# The genetic variants at the HLA-DRB1 gene are associated with primary IgA nephropathy in Han Chinese

**DOI:** 10.1186/1471-2350-13-33

**Published:** 2012-05-14

**Authors:** Yang Jiyun, Li Guisen, Zhu Li, Shi Yi, Lv Jicheng, Lu Fang, Liu Xiaoqi, Ma Shi, Jing Cheng, Lin Ying, Wang Haiyan, Wang Li, Zhang Hong, Yang Zhenglin

**Affiliations:** 1 Center for Human Molecular Biology & Genetics, Sichuan Academy of Medical Science & Sichuan Provincial People’s Hospital, 32 the First Ring Road 2 West, Chengdu, Sichuan, 617002, China; 2 Institute of Laboratory Medicine, Chengdu, China; 3 The Key Laboratory for Human Disease Gene Study of Sichuan Province, Sichuan Academy of Medical Sciences & Sichuan Provincial People’s Hospital, Chengdu, China; 4 Renal Division, Peking University First Hospital, Beijing, China; 5 Peking University Institute of Nephrology, Beijing, China; 6 Key Laboratory of Renal Disease, Ministry of Health of China, Beijing, China; 7 Renal Division, Sichuan Academy of Medical Sciences & Sichuan Provincial People’s Hospital, Chengdu, China

**Keywords:** IgA nephropathy, HLA-DRB1, Association study

## Abstract

**Background:**

Immunoglobulin A nephropathy (IgAN), an immune-complex-mediated glomerulonephritis defined immunohistologically by the presence of glomerular IgA deposits, is the most common primary glomerular disease worldwide and a significant cause of end-stage renal disease. Familial clustering of patients with IgAN suggests a genetic predisposition.

**Methods:**

In this study, 192 patients with IgAN and 192 normal controls in the Sichuan cohort and 935 patients with IgAN and 2,103 normal controls in the Beijing cohort were investigated. HLA-DRB1*01–DRB1*10 specificities were genotyped by the PCR–SSP technique in both cohorts. Based on the HLA-DRB1*04-positive results, the subtypes of HLA-DRB1*04 were analyzed using sequencing-based typing (SBT) in 291 IgAN cases and 420 matched controls.

**Results:**

The frequency of HLA-DRB1*04 in the IgAN group was significantly higher than that in the control group (0.129 vs. 0.092, *P* = 8.29 × 10^-5^, odds ratio (OR) =1.381, 95% confidence interval (CI) 1.178–1.619). Other alleles at the HLA-DRB1 locus were observed with no significant differences between the case and control groups. The dominant alleles of the HLA-DRB1*04 subtypes were DRB1*0405 in both cohorts. The frequencies of HLA-DRB1*0405 and 0403 were significantly increased in the patients compared to healthy subjects.

**Conclusion:**

HLA-DRB1*04 was significantly associated with primary IgAN in Chinese population. This result implies that HLA-DRB1 gene plays a major role in primary IgAN.

## Background

Immunoglobulin A nephropathy (IgAN), an immune-complex-mediated glomerulonephritis defined immunohistologically by the presence of glomerular IgA deposits, is the most common primary glomerular disease worldwide and a significant cause of end-stage renal disease [1]. The pathogenesis of IgAN remains poorly understood, and its treatment is limited. No consistent infectious or environmental agent has been identified as responsible for the IgA-antibody response. However, familial aggregation of patients with IgAN suggests that genetic factors contribute to the development of this disease [2]. Previous genome-wide linkage studies of multiplex families with IgAN have identified four loci with significant linkage on 6q22, 2q36, 4q26–31, and 17q12–22 [3-5]. Unfortunately, to date, no disease-specific genes linked to the loci have been identified within the linkage intervals. However, susceptibility to primary IgAN has been associated with single-nucleotide polymorphisms (SNPs) in the E-selectin and L-selectin genes, HLA-DRA, C1GalT1, and ST6GALNAC2, as well as in the PIGR gene [6-12]. Moreover, polymorphisms of the ACE and AGT genes have been associated with progression to chronic renal failure in patients with primary IgAN [13,14].

The primary function of the human leukocyte antigen (HLA) system is to regulate the immune response. MHC Class II molecules are important determinants of the IgA-mediated immune response, and the susceptibility alleles for several autoimmune diseases have now been located in the Class II region. In Caucasian patients with primary IgAN, a significant increase in the HLA-DQw7 allele frequency has been observed [15]. However, DQ alleles showed no consistent association with IgAN in different populations. In British patients, a decreased frequency of DQBI*0201 was observed, in Finnish patients a decreased frequency of DQB1*0602 was observed, and in Italian patients, no association between DQ markers and IgAN was found [16]. Earlier studies have shown that HLA-DR4 might be associated with IgAN in Japanese [17-19]. However, no association between IgAN and HLA-A, B, C, DR, and DQ was found in a Chinese Taiwanese population [20].

On the other hand, in the Italian population with a family history of primary IgAN, there was an increased incidence of HLA-DRB1*08 compared to those with sporadic primary IgAN [21]. In the present study, we investigated the association between the genetic variants at the HLA-DRB1 gene and primary IgAN in Han Chinese population.

## Methods

### Patients and controls

This study was approved by The Institutional Review Boards of the Sichuan Provincial People’s Hospital and Peking University First Hospital, China. All subjects provided informed consent before participating in the study. The diagnosis of primary IgAN was confirmed by histological and immunological examinations with renal biopsy. No clinical or serological evidence of Henoch-Schonlein purpura, systemic lupus erythematosus, alcoholic liver disease, or immunological diseases was found in any of the patients with primary IgAN. Healthy subjects were healthy volunteers with no history of renal disorder. These subjects were confirmed as being healthy by detailed clinical and laboratory examinations, such as blood pressure, urinary protein, creatinine, and urinalysis with occult blood tests. There was no familial history of primary IgAN in normal controls and cases. The Sichuan and Beijing cohorts were recruited from the renal division of Sichuan Provincial People’s Hospital and the renal division of Peking University First Hospital, respectively. In total, 192 patients with IgAN and 192 normal controls were recruited for the Sichuan cohort, and 935 patients with IgAN and 2,103 normal controls were recruited for the Beijing cohort. Clinical information about the cases and controls is listed in Table 1.

**Table 1:** Characteristics of primary IgAN cases and controls matched for age and ethnicity

Cohorts	Subject	Total number	Male	Female	Average age
Sichuan cohort	IgAN patients	192	80	112	32.07 ± 9.77
	Controls	192	62	130	63.19 ± 9.14
Beijing cohort	IgAN patients	935	498	437	31.18 ± 10.98
	Controls	2103	985	1118	49.21 ± 16.02

### HLA-DRB1 genotyping using PCR–SSP

The genotyping for HLA-DRB1 was performed using PCR–SSP as described previously [22]. The PCR amplifications were performed at 94°C or 1 min followed by 30 cycles, and the amplification conditions were different depending on HLA-DRB1*01–DRB1*10 specificities [22]. The β-actin gene was used as an internal control to ensure that the enzymatic in vitro DNA amplification process had worked in each tube.

### HLA-DRB1*04 subtypes detected by DNA sequencing-based typing (SBT)

The HLA-DRB1*04-positive samples, composed of 291 cases and 420 controls, were selected from the Sichuan cohort and the Beijing cohort by HLA-DRB1*01–DRB1*10 specificities. The subtypes of HLA-DRB1*04 were further analyzed in the HLA-DRB1*04-positive samples, as described previously [23]. The following primers were used to amplify exon 2 of the HLA-DRB1 gene: forward, 5′- CAggAAACAgCTATgACCTgAgACgCACgTTTCTTggAgCAggTTAAAC-3′; and reverse, 5′- TgTAAAACgACggCCAgTgCTYACCTCgCCKCTgCAC -3′. The PCR amplification protocols for exon 2 of the HLA-DRB1 gene were identical and consisted of an initial denaturation at 95°C for 15 min, followed by 30 cycles at 95°C for 20 s, 62°Cfor 10 s, and 72°C for 90 s. The PCR products were purified with the ExoSAP-IT® (USB Corporation) and sequenced employing ABI BigDye chemistry (Applied Biosystems). The following primers were used as sequencing primers: M13F, 5′- CAggAAACAgCTATgACC-3′; and M13R, 5′- TgTAAAACgACggCCAgT-3′. The samples were sequenced using the ABI PRISM 310 Genetic Analyzer (Applied Biosystems). uTYPE^TM^ SBT software was used to process the DNA sequence files for analysis and HLA-DRB1*04 alleles’ assignment.

### Statistical analysis

The frequency distribution of the HLA-DRB1 and HLA-DRB1*04 subtype alleles in the patient and normal control groups was compared using a Fisher’s exact test. Stratified analysis was performed using Cochran-Mantel-Haenszel method. The IgAN patients were classified into different subgroups for the purpose of studying the association of HLA-DRB1*04 alleles with clinical factors of patients by gender, blood pressure (according to the standard of hypertension of WHO-ISH in 1999), 24-h content of urine protein (≥3.5 or <3.5 g/24 h), serum creatinine level (≤120 or >120 μmol/l), hematuria (positive or negative), eGFR (≥60 or <60 ml/min/1.73 m^2^), and glomeruloscerosis (positive or negative). The strength of an association with alleles and genotypes was indicated by an odds ratio (OR) with a 95% confidence interval (CI) and *P* value. *P* value <0.05 was defined as statistically significant. SPSS version 13.0 for Windows was used for all statistical analyses.

## Results

### The frequencies of HLA-DRB1 alleles in primary IgAN patients and healthy controls

In the initial study, we tested the frequencies of each HLA-DRB1 allele in the Sichuan cohort using PCR–SSP. We found that the frequency of HLA-DRB1*04 in the IgAN group was significantly higher than that in the control group (0.143 vs. 0.073, *P* = 2.35 × 10^-3^, OR = 1.541, 95% CI, 1.130–2.100) (Table 2). Other alleles at the HLA-DRB1 locus did not show any differences between the case and control groups (Table 2). To confirm this finding, we genotyped the allele frequencies of HLA-DRB1*04 in an independent Beijing cohort, composed of 935 patients with IgAN and 2,103 normal controls. In this cohort, the frequency of HLA-DRB1*04 in the IgAN group was also significantly higher than that in the healthy control group (0.126 vs. 0.093, *P* = 2.67 × 10^-3^, OR = 1.091, 95% CI, 1.028–1.158) (Table 3). When these two cohorts were combined, the allele frequency of HLA-DRB1*04 showed a significant difference between the cases and the controls in the Han Chinese population (0.129 vs. 0.092, *P* = 8.29 × 10^-5^, OR = 1.381, 95% CI, 1.178–1.619) (Table 3).

**Table 2:** A comparison of the frequencies of HLA-DRB1 alleles in primary IgAN patients and healthy controls from the Sichuan cohort

HLA-DRB1 alleles	Case (n = 192)	Controls (n = 192)	P	OR	95% CI
DRB1*01	0.023 (9)	0.042 (16)	0.220	0.774	0.572-1.048
DRB1*03	0.055 (21)	0.047 (18)	0.743	1.088	0.769-1.538
DRB1*04	0.143 (55)	0.073 (28)	2.35 × 10^-3^	1.541	1.130-2.100
DRB1*07	0.057 (22)	0.073 (28)	0.465	0.885	0.685-1.144
DRB1*08	0.060 (23)	0.078 (30)	0.324	0.875	0.683-1.120
DRB1*09	0.158 (61)	0.151 (58)	0.842	1.031	0.844-1.258
DRB1*10	0.031 (12)	0.047 (18)	0.352	0.827	0.612-1.117
DRB1*11–12	0.073 (28)	0.089 (34)	0.508	0.904	0.713-1.147
DRB1*13-14	0.253 (97)	0.268 (103)	0.681	0.961	0.820-1.125
DRB1*15-16	0.091 (35)	0.112 (43)	0.403	0.896	0.724-1.110

OR, odds ratio; CI, confidence interval.

**Table 3:** A comparison of the frequencies of HLA-DRB1*04 in primary IgAN patients and healthy controls of different cohorts

Cohort	Case	Controls	P	OR	95% CI
Han Chinese in the Sichuan cohort^a^	0.143 (55)	0.073 (28)	2.35 × 10^-3^	1.541	1.130–2.100
Han Chinese in the Beijing cohort^a^	0.126 (236)	0.093 (392)	2.67 × 10^-3^	1.091	1.028–1.158
Both cohorts combined^b^	0.129 (291)	0.092 (420)	8.29 × 10^-5^	1.381	1.178–1.619

OR, odds ratio; CI, confidence interval; ^a^ Fisher’s exact test, ^b^Stratified analysis using Cochran-Mantel-Haenszel method; Tests of Homogeneity of the Odds Ratio:P = 0.057.

### Frequency of HLA-DRB1*04 subtypes in primary IgAN patients and healthy controls

Based on the HLA-DRB1*04-positive results, HLA-DRB1*04 subtypes were further analysed using SBT in 291 patients with IgAN and 420 matched controls. The frequency of HLA-DRB1*04 subtypes was successfully genotyped in 291 patients with IgAN and 405 controls. In the cohorts studied, the dominant alleles of the HLA-DRB1*04 subtypes were DRB1*0405. The frequencies of HLA-DRB1*0405 and 0403 were significantly increased in the patients compared to healthy subjects (Table 4).

**Table 4:** A comparison of the frequencies of HLA-DRB1*04 subtypes in primary IgAN patients and healthy controls of Han Chinese descent

HLA-DRB1 alleles	Case (n = 935 × 2)	Controls (n = 2088 × 2)	P	OR	95% CI
DRB1*0401	0.011 (21)	0.0010 (43)	0.786	1.028	0.866–1.221
DRB1*0402	0.003 (5)	0.002 (8)	0.556	1.123	0.730–1.726
DRB1*0403	0.029 (54)	0.014 (60)	2.088 × 10^-4^	1.318	1.107–1.570
DRB1*0404	0.005 (10)	0.007 (28)	0.601	0.937	0.774–1.134
DRB1*0405	0.067 (125)	0.037 (153)	5.225 × 10^-7^	1.267	1.138–1.411
DRB1*0406	0.026 (49)	0.021 (89)	0.263	1.073	0.947–1.215
DRB1*0407	0.004 (7)	0.002 (9)	0.284	1.229	0.797–1.893
DRB1*0408	0.002 (3)	0.002 (8)	1.000	0.950	0.661–1.364
DRB1*0409	0	0.009 (4)	0.318	-	-
DRB1*0410	0.006 (11)	0.002 (9)	0.027	1.537	0.946–2.495
DRB1*0411	0.010 (3)	0	0.030	-	-
DRB1*0419	0.001 (2)	0	0.096	0.416	0.381–0.455
DRB1*0420	0.0005(1)	0.0002(1)	0.523	1.382	0.345–5.525

OR, odds ratio; CI, confidence interval.

### The association of HLA-DRB1*04 alleles and its subtypes with clinical factors in primary IgAN patients

The IgAN patients were classified into different subgroups according to clinical factors. We analysed the association of HLA-DRB1*04 alleles with clinical factors. However, our data showed no significant association between clinical factors and the frequency of the HLA-DRB1*04 alleles and its subtypes in primary IgAN patients (Table 5).

**Table 5:** The association of HLA-DRB1*04 alleles with clinical and pathological parameters of primary IgAN patients

		HLA-DRB1*04	P	OR	95% CI
Gender (n = 1104)	Female	0.133 (143)	0.850	1.015	0.809-1.146
	Male	0.130 (157)			
SCr (umol/L) (n = 1079)	≤120	0.136 (229)	0.705	1.051	0.830-1.331
	>120	0.129 (62)			
Hematuria (n = 1061)	No	0.125 (19)	0.806	0.994	0.961–1.028
	Yes	0.135 (266)			
Upro (g/24Hr) (n = 1056)	≤3.5	0.138 (233)	0.875	1.025	0.798–1.316
	>3.5	0.134 (57)			
Blood pressure (n = 1076)	Normal	0.143 (171)	0.228	1.095	0.946–1.267
	Hypertension	0.125 (120)			
eGFR, ml/min/1.73 m^2^ (n = 1079)	≤60	0.130 (241)	0.784	0.964	0.711–1.307
	>60	0.135 (41)			
Glomeruloscerosis (n = 1079)	No	0.124 (201)	0.120	0.844	0.691–1.032
	Yes	0.151 (81)			

OR, odds ratio; CI, confidence interval.

## Discussion

Previous studies have demonstrated that the HLA loci are associated with IgAN in different populations but not consistent with any alleles [1,15-20,24]. A family-based and case–control association study using the genome-wide association study (GWAS) approach suggested that the HLA region contains the strongest common susceptibility alleles of primary IgAN in a small European cohort [25]. HLA Imputation Analysis shows that the HLA-B, DRB1, DQA, and DQB loci are associated with IgAN, and the strongest association was observed at the HLA-DQ locus in the study [25]. Recently, a GWAS for primary IgAN identified three independent loci in the major histocompatibility complex in a cohort of 3,144 primary IgAN cases of Chinese and European ancestry. The study showed that 27 SNPs exceeding genome-wide thresholds for significance were located in a 0.54-Mb interval within the MHC. Imputation of classical HLA alleles showed that the strongest association was located within a ~170-kb interval that includes HLA-DRB1, HLA-DQA1, and HLA-DQB1 [26]. This further indicated that the HLA-DRB1 region is one of the major primary IgAN susceptibility loci.

The HLA-DRB1 locus is, by far, the most polymorphic MHC class II locus and has more than 540 alleles [27]. Previous studies have found a significant association between IgAN and the HLA-DRB1 alleles in a Japanese population [17,19,28]. Recently, a study showed that HLA-DRB1 polymorphisms were related to the occurrence and disease progression of Han Chinese patients with primary IgAN. HLA-DRB1*140501 was reported to be a susceptible allele, and HLA-DRB1*070101 was reported to be a resistant allele. HLA-DRB1*030101 may serve as a predictor of disease progression and renal damage of primary IgAN in Han Chinese [29]. However, previous studies did not substantiate the conclusion that HLA-DRB1 was significantly associated with IgAN because a small number of samples were included in these studies and there was no association between the HLA-DR antigen and primary IgAN in Taiwanese Chinese [20]. In our studies, all of these samples were pre-typed at a low resolution using PCR –SSP, which provided information on the HLA-DRB1 alleles at serological recognition. Then, we typed the HLA-DRB1*04 subtype alleles using a high resolution sequence-based HLA-DRB1 typing of Exon 2. Compared with HLA-DRB1 typing using the SBT approach for these samples, it took less time and money to determine the allele frequencies of HLA-DRB1 using a two-stage typing approach combined with PCR-SSP and SBT. We further confirmed that the genetic variants at the HLA-DRB1 gene were significantly associated with IgAN in a large Han Chinese cohort composed of 1,127 cases and 2,295 controls. However, since our approach does not distinguish between the alleles DRB1*11 and *12, DRB1*13 and *14 and DRB1*15 and *16, it could be missing the true associations between other HLA-DRB1 alleles as well. In addition, the IgAN GWAS study demonstrated a strong protective effect of the DRB1*15 allele (OR=0.61, *P*=10-6) and potentially of other DRB1 alleles [26].

As for diseases of polygenic inheritance, there are different disease susceptibility genes in different populations. Primary IgAN, defined as predominant IgA mesangial deposits in the absence of clinical or laboratory evidence of other associated systemic diseases, has also been proposed to include at least two distinct clinical entities (those with and without macroscopic hematuria) [1,30,31], which may be determined by different susceptibility genes. Therefore, heterogeneity between populations may account for some of the controversy in HLA associations with IgAN.

The allelic frequency and diversity of HLA-DRB1 among different populations emphasizes the need for research using matched patient and control groups in genetic association studies. Some reports indicate that the population of Metropolitan Beijing, in the northern part of China, includes a mixture of individuals from both the northern and central provinces. However, these reports have also found that there are few people from the southern region[32]. In addition, the reports indicate that the population mix of the Sichuan province, considered representative of southern China, could in fact have been affected by the mass migrations during the late Ming and early Qing dynasties. At those times, people came to Sichuan from both the southern and the central regions, such as Hunan and Hubei[32]. As a result, the cases and controls for our studies have been recruited through geographic matching, to eliminate the adverse impact of stratification. We evaluated the allelic frequency of HLA-DRB1 in the Sichuan cohort (192 cases and 192 controls) and the Beijing cohort (935 cases and 2,103 controls). Subsequently, we analyzed the Sichuan and Beijing cohorts together and confirmed a strong association with primary IgAN susceptibility, that the allele frequency of HLA-DRB1*04 was significantly associated with primary IgAN susceptibility. HLA-DRB1*0405 and 0403 were the susceptible allele of pIgAN patients in Han Chinese population. However, there is no significant association between clinical factors and the frequency of the HLA-DRB1*04 alleles and its subtype in primary IgAN patients.

In summary, this study showed that the HLA-DRB1*04 had a strong association with primary IgAN in a Chinese population. In our disease association studies, all of these samples were pre-typed at a low resolution using PCR –SSP, which may limit our ability to detect rare alleles of HLA DRB1. The primary technology used to detect rare alleles of HLA-DRB1 is the high resolution SBT, which could contribute to the proper identification of susceptible or resistant alleles of IgAN. HLA-DRB1 has been shown to be associated with many autoimmune diseases, however, the mechanism of the HLA-DRB1 causing IgAN is poorly understood. Thus further research exploring the pathogenesis of HLA-DRB1 with IgAN is needed.

## Conclusion

HLA-DRB1*04 was significantly associated with primary IgAN in Chinese population. HLA-DRB1*0405 and 0403 were the susceptible allele of pIgAN patients in Han Chinese population. However, there is no significant association between clinical factors and the frequency of the HLA-DRB1*04 alleles and its subtype in primary IgAN patients.

## Competing interests

The authors declare that they have no competing interests.

## Authors’ contributions

JY carried out genotyping assays, statistical analyses, and drafted the manuscript. GL and LZ performed the statistical analysis of the genotypic data. YS, JL, FL, XL, SM, JC, YLHW, and LW collected samples. HZ and ZY conceived of the study and helped to critically draft the manuscript. All authors read and approved the final manuscript.

## Pre-publication history

The pre-publication history for this paper can be accessed here:

http://www.biomedcentral.com/1471-2350/13/33/prepub
